# Composite Selection Signals for Complex Traits Exemplified Through Bovine Stature Using Multibreed Cohorts of European and African *Bos taurus*

**DOI:** 10.1534/g3.115.017772

**Published:** 2015-04-30

**Authors:** Imtiaz A. S. Randhawa, Mehar S. Khatkar, Peter C. Thomson, Herman W. Raadsma

**Affiliations:** Reprogen–Animal Bioscience Group, Faculty of Veterinary Science, The University of Sydney, Camden, 2570, New South Wales, Australia

**Keywords:** body size, outbred populations, polygenic traits, signatures of selection, stature genes

## Abstract

Understanding the evolution and molecular architecture of complex traits is important in domestic animals. Due to phenotypic selection, genomic regions develop unique patterns of genetic diversity called signatures of selection, which are challenging to detect, especially for complex polygenic traits. In this study, we applied the composite selection signals (CSS) method to investigate evidence of positive selection in a complex polygenic trait by examining stature in phenotypically diverse cattle comprising 47 European and 8 African *Bos taurus* breeds, utilizing a panel of 38,033 SNPs genotyped on 1106 animals. CSS were computed for phenotypic contrasts between multibreed cohorts of cattle by classifying the breeds according to their documented wither height to detect the candidate regions under selection. Using the CSS method, clusters of signatures of selection were detected at 26 regions (9 in European and 17 in African cohorts) on 13 bovine autosomes. Using comparative mapping information on human height, 30 candidate genes mapped at 12 selection regions (on 8 autosomes) could be linked to bovine stature diversity. Of these 12 candidate gene regions, three contained known genes (*i.e.*, *NCAPG-LCORL*, *FBP2-PTCH1*, and *PLAG1-CHCHD7*) related to bovine stature, and nine were not previously described in cattle (five in European and four in African cohorts). Overall, this study demonstrates the utility of CSS coupled with strategies of combining multibreed datasets in the identification and discovery of genomic regions underlying complex traits. Characterization of multiple signatures of selection and their underlying candidate genes will elucidate the polygenic nature of stature across cattle breeds.

Genomes that have experienced selective pressure due to their functional role in phenotypic diversity contain unique patterns of genetic diversity called signatures of selection (Oleksyk *et al.* 2010). Recent progress in molecular genetics has provided powerful tools to generate and analyze genetic polymorphism data (Lenstra *et al.* 2011; Hancock and Di Rienzo 2008; Crisci *et al.* 2012). Consequently, genome-wide scans to detect signatures of selection have been conducted in several species to understand the role of molecular variants in shaping their genetic diversity (Gibbs *et al.* 2009; Ramey *et al.* 2013; Kijas *et al.* 2012; Zhang *et al.* 2012; Petersen *et al.* 2013; Qanbari *et al.* 2014; Utsunomiya *et al.* 2013; Akey *et al.* 2010).

Footprints of sustained selection in livestock are generally diverse and complex. It has been a great challenge to differentiate between the neutrally (random drift) and rapidly (selection) evolving traits (Hohenlohe *et al.* 2010). In general, signatures of selection are identified from the patterns of DNA variation without recorded phenotypic information on populations undergoing investigation. Nonetheless, the signatures of selection detected between populations are often indicative of segregating regions of functional mutations underlying divergently selected quantitative traits (Hayes *et al.* 2008, 2009). In complex genomes such as mammalian species, the functional mutations and their systematic patterns of polymorphism exist in multiple locations. Identification of the trait-specific genomic regions requires comprehensive phenotypic and genomic data of large populations to detect causal effects of selection (Decker *et al.* 2012). Despite using known mapping approaches, it has often been difficult to localize historical selection events for complex traits (Kemper *et al.* 2014). A number of tests are available to detect candidate regions under selection, which often provide differing results from the same genomic dataset (Qanbari *et al.* 2011; Crisci *et al.* 2012). Combining several tests with complementary characteristics can improve the ability to localize the candidate regions under selection (Utsunomiya *et al.* 2013; Grossman *et al.* 2010).

Recently, we developed a composite selection signal (CSS) test (Randhawa *et al.* 2014), a weighted index of signatures of selection from multiple estimates, to identify genomic regions under selection in domestic species. CSS is a nonparametric procedure that uses fractional ranks of constituent tests and it does not depend on their distributional assumptions. Hence, as compared to the previous approaches of combining multiple selection tests that have limitations to incorporate only the statistics with *p*-values (Utsunomiya *et al.* 2013; Grossman *et al.* 2010), CSS has an appealing feature to combine the evidence of historical selection from any selection test. We demonstrated that CSS is a robust method to identify loci for across-population genetic diversity of genomic regions, linking them to target phenotypes of monogenic traits under selective pressure (Randhawa *et al.* 2014). CSS can be extended to complex traits. It is expected that co-selection on multiple genomic loci underling a complex trait can be captured in the form of CSS by combining the signals detected from the patterns of population differentiation, derived allele frequency, and the extended haplotype homozygosity.

The quantitative trait loci (QTL) and signatures of selection mapped at the candidate regions suggested the role of underlying genes in diversity of stature across various breeds (Druet *et al.* 2013; Ramey *et al.* 2013; Karim *et al.* 2011; Nishimura *et al.* 2012; Kemper *et al.* 2014).

Diversity in bovine stature can potentially be used as a model to demonstrate the application of new approaches, such as CSS, for the detection of selection for complex traits in cattle, given that the trait can be easily measured and data are available in a wide range of worldwide breeds. Notably, stature is a trait strongly influenced by domestication (Karim *et al.* 2011) and has a known history of selective pressure over time, both negative and positive (Ajmone-Marsan *et al.* 2010; Fortes *et al.* 2013). Stature (or adult height) is a complex trait under polygenic control with high heritability in many mammalian species including cattle (Kemper and Goddard 2012; Lettre 2011; Lanktree *et al.* 2011). Genetic architecture of height has been extensively investigated to identify genes with major effects across the genome of multiple species, although the leading model has been the human genome (Lettre *et al.* 2008; Sanna *et al.* 2008; Shiura *et al.* 2009; Tetens *et al.* 2013; Deng *et al.* 2013; Lanktree *et al.* 2011). To date, only a few genes responsible for stature, including body size traits, have been reported in cattle from genome-wide association studies (GWAS) (Pryce *et al.* 2011; Fortes *et al.* 2013; Li *et al.* 2013; Hoshiba *et al.* 2013; Bolormaa *et al.* 2014).

Two major candidate gene regions, *NCAPG-LCORL* and *PLAG1-CHCHD7*, on chromosome 6 and chromosome 14, respectively, have been linked to stature in European breeds of cattle. The *NCAPG* and *LCORL* genes are strong candidates for growth and height-related traits in multiple species of mammals, including cattle (Gudbjartsson *et al.* 2008; Weedon *et al.* 2008; Soranzo *et al.* 2009; Lango Allen *et al.* 2010; Okada *et al.* 2010; Pryce *et al.* 2011; Sovio *et al.* 2009). This candidate gene region has frequently shown strong signatures of selection in multiple breeds of cattle (Barendse *et al.* 2009; Druet *et al.* 2013; Flori *et al.* 2009; Gibbs *et al.* 2009; Kemper *et al.* 2014; Mancini *et al.* 2014; Pintus *et al.* 2013; Porto-Neto *et al.* 2013; Rothammer *et al.* 2013; Stella *et al.* 2010; Qanbari *et al.* 2014; Perez *et al.* 2014). Several polymorphic variants at this locus have been identified in GWAS and QTL studies for wither height in horse (Tetens *et al.* 2013; Signer-Hasler *et al.* 2012), and growth, skeletal, carcass, and stature traits in cattle (Lindholm-Perry *et al.* 2011; Pryce *et al.* 2011; Hoshiba *et al.* 2013; Nishimura *et al.* 2012; Bolormaa *et al.* 2014). The *PLAG1-CHCHD7* locus harbors nine candidate genes and has been associated with height in humans (Gudbjartsson *et al.* 2008; Soranzo *et al.* 2009; Okada *et al.* 2010; Lettre *et al.* 2008; Lango Allen *et al.* 2010) and stature in cattle (Nishimura *et al.* 2012; Pryce *et al.* 2011; Karim *et al.* 2011; Fortes *et al.* 2013; Bolormaa *et al.* 2014). This locus was also identified as a candidate region based on selective sweeps in several cattle breeds that have been under strong selection for body size (Druet *et al.* 2013; Ramey *et al.* 2013; Kemper *et al.* 2014). However, known genes account only for a small proportion of the phenotypic variation in bovine stature (Kemper and Goddard 2012). Comparative mapping provides additional evidence that some orthologous genes of human and cattle affect stature in both species (Pryce *et al.* 2011). Therefore, the comparative mapping of genes identified in human GWAS can be used as supporting evidence to elucidate additional candidate gene regions in cattle (Visscher and Goddard 2011).

Selective pressure on phenotypically similar populations may have acted on the same causal variants in analogous genes (Kijas *et al.* 2012; Stella *et al.* 2010; Pickrell *et al.* 2009). Therefore, a multibreed panel by combining phenotypically alike breeds, and comparing such multibreed groups for contrasting phenotypes can maximize the detectable signatures of selection for bovine stature (Barendse *et al.* 2009; Stella *et al.* 2010; Kemper and Goddard 2012; Visscher and Goddard 2011).

This study applies the CSS method to identify candidate genomic regions harboring genes responsible for bovine stature, a polygenic complex trait. Cohorts of contrasting phenotypes were investigated in multibreed panel comparisons using morphological information on wither height of 55 worldwide breeds of European and African *Bos taurus*. Finally, we compiled orthologous genes from several GWAS on human height and mapped them on the bovine genome assembly (UMD3.1) to explicate the significant genomic regions in cattle defined by CSS.

## Materials and Methods

### Genotypic data

In total, 1106 animals of 55 breeds were genotyped with an Illumina BovineSNP50 chip assay (Supporting Information, Table S1, Table S2). Information regarding DNA samples and SNP genotypes were previously reported (Decker *et al.* 2009; Gautier *et al.* 2009, 2010). The duplicate samples across multiple datasets of cattle were identified and removed based on an IBS matrix computed using PLINK (Purcell *et al.* 2007). The IBS matrix estimates the genetic relationship based on genome-wide SNPs similarity. A quality control was implemented to retain SNPs that can be mapped on UMD3.1 bovine assembly and have minor allele frequency (MAF) ≥0.01 (Table S3). Ancestral and derived allelic phases of these SNPs were determined based on information on several closely related out-group species, including bison, buffalo, and yak (Decker *et al.* 2009; Matukumalli *et al.* 2009). Imputation of missing genotypes and haplotype phasing were performed with BEAGLE 3.3 (Browning and Browning 2009). Analyses of signatures of selection for European and African cohorts were performed using polymorphic and phased SNPs within each cattle type.

### Phenotypic data

Bovine stature is generally defined as height at the withers; however, this information was not directly available on the genotyped animals of the selected breeds. Therefore, in this study, for a broader perspective of phenotypic characterization, wither heights of 47 European *Bos taurus* breeds (listed in Table S1) and 8 African *Bos taurus* breeds (Table S2) reported by 52 countries were acquired from the online database (DAD-IS 2014) of the Food and Agriculture Organization (FAO). FAO data for the bovine stature are available as the country-wise breed averages for male and female height at withers. Availability of breed averages for wither height ranged from a single country (*i.e.*, country of breed origin or country of DNA sampling) to many countries (*n* = 37). Breed-wise as well as overall estimates of median, upper quartile, and lower quartile of whither height were computed to categorize breeds into phenotypically contrasting cohorts (see below). For this purpose, first, country-wise stature data for each breed were prepared from the averages of male and female withers heights (cm). Second, if a breed was represented by a single country, then the country’s average stature for the breed was used to represent the breed; otherwise, the median stature from multiple countries (based on the FAO data available as country-wise average) was used.

### Multibreed cohorts of contrasting stature

The contrasting cohorts of multiple breeds, within European (Table S1) and African (Table S2) breed types, were defined for analyses following the expectation that identification of plausible signatures of selection for stature would require a relatively stringent separation of breeds and sufficient sample sizes in each cohort. Therefore, a stringent strategy was adopted to construct two cohorts of contrasting phenotypes, *i.e.*, the large and small stature cohorts (Figure S1). The individual breeds that have their median stature above the overall upper quartile and below the overall lower quartile were categorized into the large and the small cohorts, respectively. Moreover, the large cohort’s breeds (represented by several countries) are further restricted to have their individual lower quartiles above the overall median. Similarly, the small cohort’s breeds (represented by several countries) have their individual upper quartiles below the overall median. Separate analyses to identify signatures of selection were performed to compare the large and small cohorts of European and African breeds.

Previously, we have shown that cohort composition had minimal effect on the computation of composite selection tests when the breeds with small sample size (*n* < 10 and *n* < 20) were excluded (Randhawa *et al.* 2014). Further, in this study, we also assessed the effect of cohort composition comprising European breeds of cattle, specifically whether a few breeds with large sample size (*n* > 20) can disproportionately affect the analyses. To this effect we performed additional analyses by limiting the sample size (*n* ≤ 10 and *n* ≤ 20) per breed. These results were compared to those from the original cohorts, where we included all available samples from each breed and the constituent and composite selection tests were computed by analyzing the European large and small cohorts against each other.

### Composite selection signal

The detailed methods of computing composite selection signal (CSS) are provided by Randhawa *et al.* (2014). Briefly, the CSS statistics were computed at each locus by combining three popular constituent selection methods, namely, change in allele diversity by *F*_ST_ (Weir and Cockerham 1984) across-population extended haplotype homozygosity (XP-EHH) test (Sabeti *et al.* 2007), and change in derived allele frequencies (ΔDAF) test (Grossman *et al.* 2010). *F*_ST_ statistics were computed as the differentiation index between the large and small cohorts. XP-EHH and ΔDAF statistics were computed for the large and small cohorts such that, by analyzing the large (as selected) cohort against the small (as nonselected) cohort and vice versa. The composite statistics (CSS) were computed as follows:

1. For each constituent method, test statistics were ranked (1, ..., *n*) genome-wide on *n* SNPs.
2. Ranks were converted to fractional ranks (*r*′) (between 0 and 1) by 1/(*n* + 1) through *n*/(*n* + 1).
3. Fractional ranks were converted to *z*-values as *z* = Φ^-1^(*r*′) where Φ^-1^(⋅) is the inverse normal cumulative distribution function (CDF).
4. Mean *z* scores were calculated by averaging *z*-values across all constituent tests at each SNP position and *p*-values were directly obtained from the distribution of means from a normal *N*(0, *m^−^*^1^) distribution where *m* is the number of constituent test statistics.
5. Logarithmic (−log_10_ of *p*-values) of these mean *z*-values were declared as CSS and were plotted against the genomic positions to identify the significant selection signals.
6. To reduce spurious signals, the empirical CSS scores were smoothed by averaging values within 0.5 Mb sliding windows on both sides at each SNP. The top 0.1% of smoothed CSS scores was used to declare a significant SNP relative to the rest of the genome.

### Genomic regions and genes under selection

Following a similar approach of Gibbs *et al.* (2009) and Kijas *et al.* (2012), significant genomic regions were defined as those that harbor at least one significant SNP (top 0.1%) flanked by a set of five or more adjoining SNPs in the top 1%. The genomic positions of the significant regions were defined by the first and last of the top 1% SNP in the cluster. In addition, multiple clusters localized in close proximity (∼1 Mb) were considered as a single significant genomic region.

The genes underlying the significant regions and surrounding locations (∼1 Mb of cluster boundaries) were listed. These putative genes were searched for evidence of association or signatures of selection for stature within other mammalian species. For this purpose, the records of orthologous genes for the major GWAS reports on human height (Gudbjartsson *et al.* 2008; Lango Allen *et al.* 2010; Lanktree *et al.* 2011; Lettre *et al.* 2008; Okada *et al.* 2010; Weedon *et al.* 2008; Soranzo *et al.* 2009; Kim *et al.* 2010) were mapped on UMD3.1 bovine genome assembly by matching the gene names] (Table S4).

In total, 268 orthologous genes were successfully mapped on the bovine autosomes (BTA) and were located in 134 genomic locations, termed as “candidate gene regions” here and defined by the consecutive orthologous genes located on a chromosome within 1 Mb distance from each other. The number of genes per region ranged from one to nine, with an average size of 355 kb. Hence, the 134 regions harboring genes for human stature covered 47.50 Mb (1.9%) of the bovine genome. In addition, after adding 1 Mb surrounding of each candidate gene region, these regions cover 7% of the bovine genome. Hence, finding a candidate gene carrying CSS region out of those 134 regions as expected by chance is equal to 7%.

To compare whether the proportion of identified regions linked to the bovine stature in various analyses was by chance, we performed a permutation test whereby the animals were randomly assigned to two cohorts under a no-selection model. Sample sizes of the permuted cohorts were equal to the samples sizes of real cohorts and samples from individual breeds within cohorts were not weighted. Five sets of independently permuted cohorts, separately for European and African breeds, were analyzed by CSS and the results were compared against the empirical cohorts.

Finally, signatures of selection identified by CSS in candidate gene regions were further classified as previously reported in cattle or novel to the species. Signatures of selection outside of the candidate gene regions were considered as regions of interest that may be putatively related to stature.

## Results

### Genotypic data after quality control

Genotypic data passing quality control consisted of 38,033 SNPs that were filtered after combining the available data on various cattle breeds from Decker *et al.* (2009) and Gautier *et al.* (2009, 2010) (see Table S3 to access raw datasets). The retained SNPs had MAF ≥0.01, known ancestral and derived alleles, and mapped on 29 autosomes covering 2,512.08 Mb of UMD3.1 bovine genome assembly. The mean (median) distance between consecutive SNPs on the bovine autosomes was 65.52 kb (64.54 kb), ranging from 2.22 kb to 1.44 Mb (Table S3). Finally, data subsets of 37,218 and 33,024 informative SNPs were analyzed to detect signatures of selection within European and African cattle, respectively.

### Classification of breeds into phenotypically contrasting cohorts

**Figure 1:**
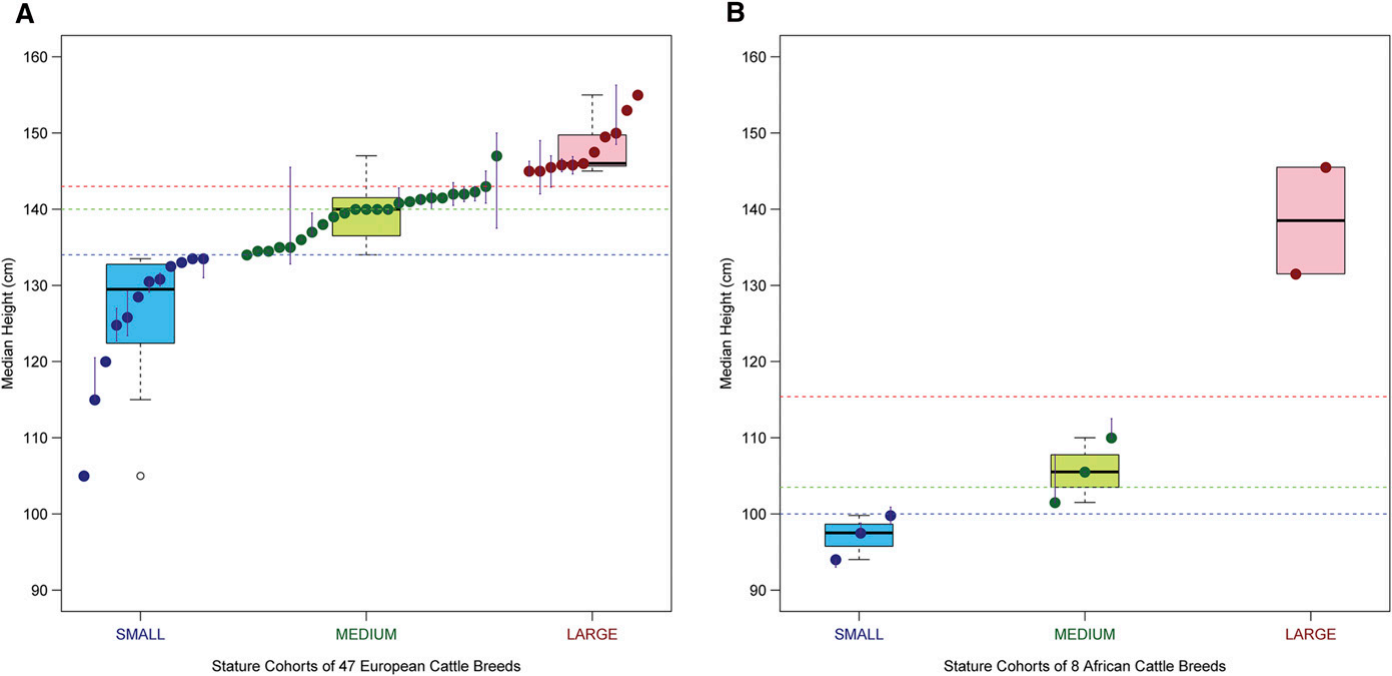
Distribution of the wither height (cm) of the 47 European (A) and eight African (B) *Bos taurus* breeds. Each colored dot represents the median of a breed’s stature using FAO data from the multiple countries and the vertical error bars range between the upper and lower quartiles. Several breeds are represented from a single country; therefore, they do not have error bars. The horizontal dashed red, green, and blue lines, respectively, represent the overall upper quartile, median, and lower quartile of all breeds’ data. The red, green, and blue dots represent cattle breeds categorized in the large, medium, and small stature cohorts, respectively (see Figure S1). Boxplots show distribution of breed median data for stature within each cohort.

From the FAO data (DAD-IS 2014), the overall lower quartile (Q1), median, and upper quartile (Q3) for the combined values for wither height of 47 European breeds were 134 cm, 140 cm, and 143 cm, respectively (Figure 1A). In comparison, the median wither height for the European large (11 breeds) and European small (12 breeds) stature cohorts were 146.8 cm and 129.5 cm, respectively (Table S1, Figure 1A). The minimum median (145 cm of Charolais and Montbeliarde breeds) of the large cohort and the maximum median (133.5 cm of Finnish Ayrshire and Belted Galloway breeds) of the small cohort values were separated by 11.5 cm.

Similarly using the FAO data for the eight African breeds, overall Q1, median, and Q3 for wither heights calculated were 100 cm, 106 cm, and 115 cm, respectively (Figure 1B). The median wither heights for the African large (two breeds) and African small (three breeds) stature cohorts were 138.5 cm and 97.5 cm, respectively (Table S2). The separation of closest medians at the tails of the two African cohorts was 31.7 cm.

Overall, the distribution of breed-wise data (Figure 1) shows that implementing the stringent grouping strategy resulted in a clear separation (contrast) of wither height (stature) between the large and the small cohorts of the European and African categories.

### Significant CSS

In the analysis of the European and African cohorts, 38 and 33 SNPs represented the top 0.1%, respectively, based on ranks of smoothed CSS, and these were used to map the significant genomic regions. The number of top 0.1% SNPs within each significant region ranged from 1 to 20 (Figure S2). The number of significant regions ranged from four to nine (Table 1). The number of top SNPs per region and region size was highest in the European cohorts and lowest in the African cohorts (Figure S2). Median (mean) size of the significant regions was 920 kb (1.20 Mb), ranging from 410 kb to 3.46 Mb. Moreover, merging two neighboring regions of European small cattle cohort on BTA-3 increased the maximum region length to 6.34 Mb.

**Table 1:** Summary of significant signatures of selection and the candidate regions containing stature-associated genes

Candidate Populations		Reference Population***^a^***		
Categories	Cohorts	Small	Large	Total Regions
**European**	Large	4 (4)***^b^***	—	9 (7)
	Small	—	5 (3)	
**African**	Large	9 (2)	—	17 (5)
	Small	—	8 (3)	
**Overall**				26 (12)

**Figure 2:**
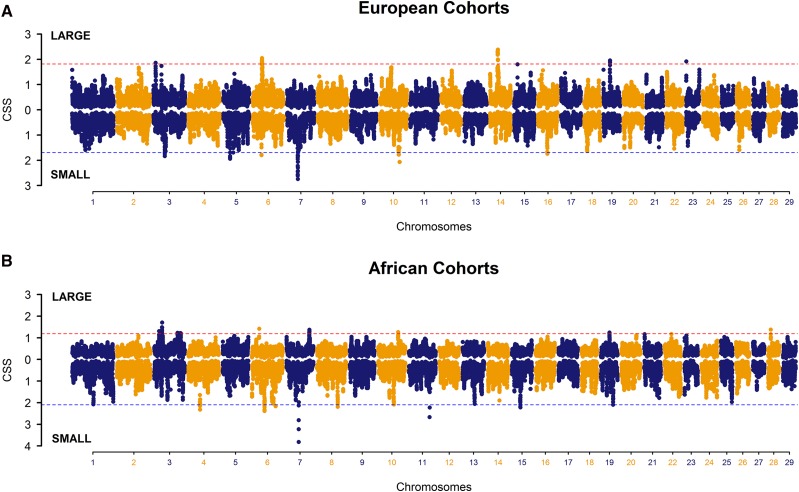
Manhattan plots of genome-wide (smoothed) composite selection signals (CSS) in the large and small cohorts of (A) European and (B) African breeds. Each cohort was compared against the other as a reference population, as shown in each Manhattan plot for positive and negative CSS scores above or below the dashed lines. Dashed lines (red for positive CSS and blue for negative CSS) indicate the cut-off at the top 0.1% of the genome-wide smoothed CSS distribution.

Complete CSS results are given in Figures and Supplementary Tables for European cohorts (Figure 2A, Table S5) and African cohorts (Figure 2B, Table S6). Significant signatures of selection (top 0.1% of CSS) and some candidate genes (complete list in Table 2) within each region are shown in Figure 4.

a Reference populations for candidate European cohorts were the contrasting European cohorts; similarly, African cohorts were compared against contrasting African cohorts.

b Each cell shows number of significant regions identified by analyzing the candidate population against the contrasting reference population. The numbers within parentheses are significant regions that harbor candidate genes associated with stature.

### CSS in European cattle cohorts

Genome-wide distribution of the smoothed CSS (−log_10_*p*) for the large and small cohorts of European cattle identified nine genomic regions carrying clusters of significant SNPs **(**Figure 2A). A complete list of putative regions identified in the two cohorts of European *Bos taurus* is provided in Table S5. In the large cohort, the selected regions were identified on BTA-3, BTA-6, BTA-14, and BTA-19. In the small cohort, the selected regions were located on BTA-3, BTA-5, BTA-7, BTA-10, and BTA-16.

Figure S4 shows a genome-wide comparison for the distribution of significant CSS peaks between the original results (Figure 2A) and three additional analyses—performed to evaluate composition of European cohorts given the variable sample sizes of cattle breeds. Overall, high concordance was observed between the significant peaks in cohort-wise analyses using all available individuals (original), with a maximum of 20 and 10 individuals per breed. For comparison, the significant regions were considered valid if found within the top 1% significant threshold. Notably, the significant regions detected in original analyses (Table S5) were found above the top 1%, with the only exception being a region on BTA-19 in the European large cohort being not detected with a maximum of 10 individuals per breed. Overall, the impact of varying the number of animals per breed had negligible impact on the CSS profile. Hence, we propose that the maximum number of animals should be used when possible.

### CSS in African cattle cohorts

The Manhattan plot for African cohorts shows smoothed CSS results from the intercohort comparisons (Figure 2B). We observed that the genome-wide CSS peaks were less pronounced in African cohorts compared with European breeds. A complete list of 17 regions identified in the two cohorts of African *Bos taurus* is provided in Table S6. Overall, nine regions (on BTA-3, BTA-7, BTA-10, BTA-19, and BTA-22) were identified carrying selection signatures in the African large cohort and eight regions (on BTA-4, BTA-6, BTA-7, BTA-8, BTA-10, BTA-11, BTA-15, and BTA-19) were identified in the African small cohort.

### Genomic regions and candidate genes for stature

Table 1 shows a summary of the significant regions in each cohort with comparison to respective reference populations within the European and African categories. It also shows the number of regions harboring candidate genes associated with stature, which were found in human GWAS (Table S4). Overall, out of 9 and 17 significant genomic regions within the European and African cohorts, 7 (77.8%) and 5 (29.4%) were co-located with candidate gene regions that harbor stature-associated genes. The remaining 2 and 12 significant genomic regions in European and African cohorts, respectively, were identified outside the known candidate gene regions. Overall, 46% (*n* = 12 out of 26) of all significant CSS were co-located with candidate gene regions, this proportion is much higher as compared to the 7% expected by chance alone as described above (Figure S3).

**Figure 3:**
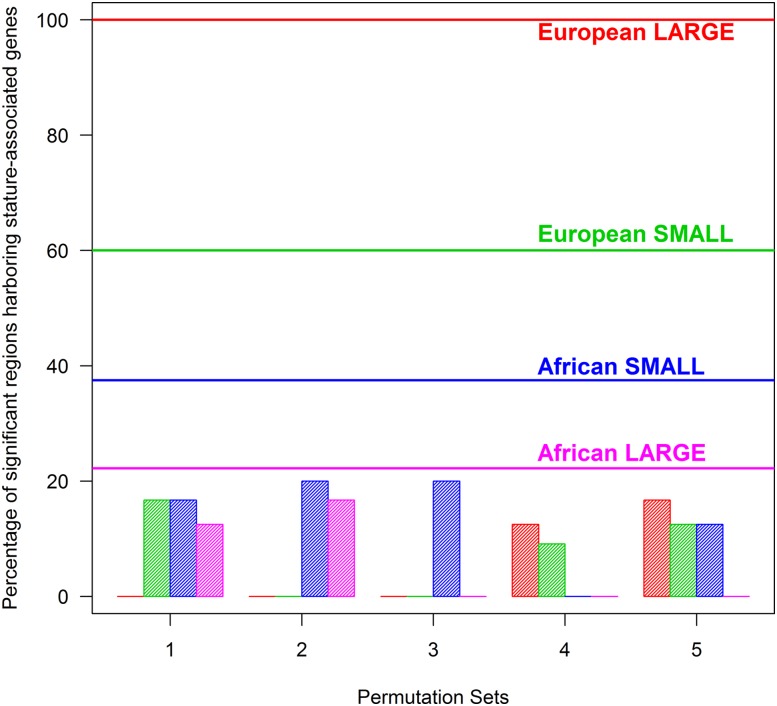
Comparison of empirical and permuted CSS scores for localization of significant regions harboring stature-associated genes. Horizontal lines, labeled with cohorts (European large = 100%, European small = 60%, African small = 37.5%, and African large = 22.2%), indicate the percentage of significant genomic regions from each cohort that localized the stature associated genes. Vertical bars show the distribution of significant regions (%) harboring stature-associated genes in five sets of permutation cohorts that mimicked the empirical cohorts in matching colors.

Figure 3 shows a summary comparison of empirical and permuted cohort results. Overall, 8.3% of the significant regions based on permuted CSS in five sets (*i.e.*, 20 permuted cohorts) contained stature-associated loci (Figure 3) that was in agreement with the 7% expected by chance (Figure S3). On average, only 6.7% and 9.8% of significant regions of permuted CSS from European and African cohorts had stature-associated genes as compared to 77.8% and 29.4% of empirical regions, respectively.

**Figure 4:**
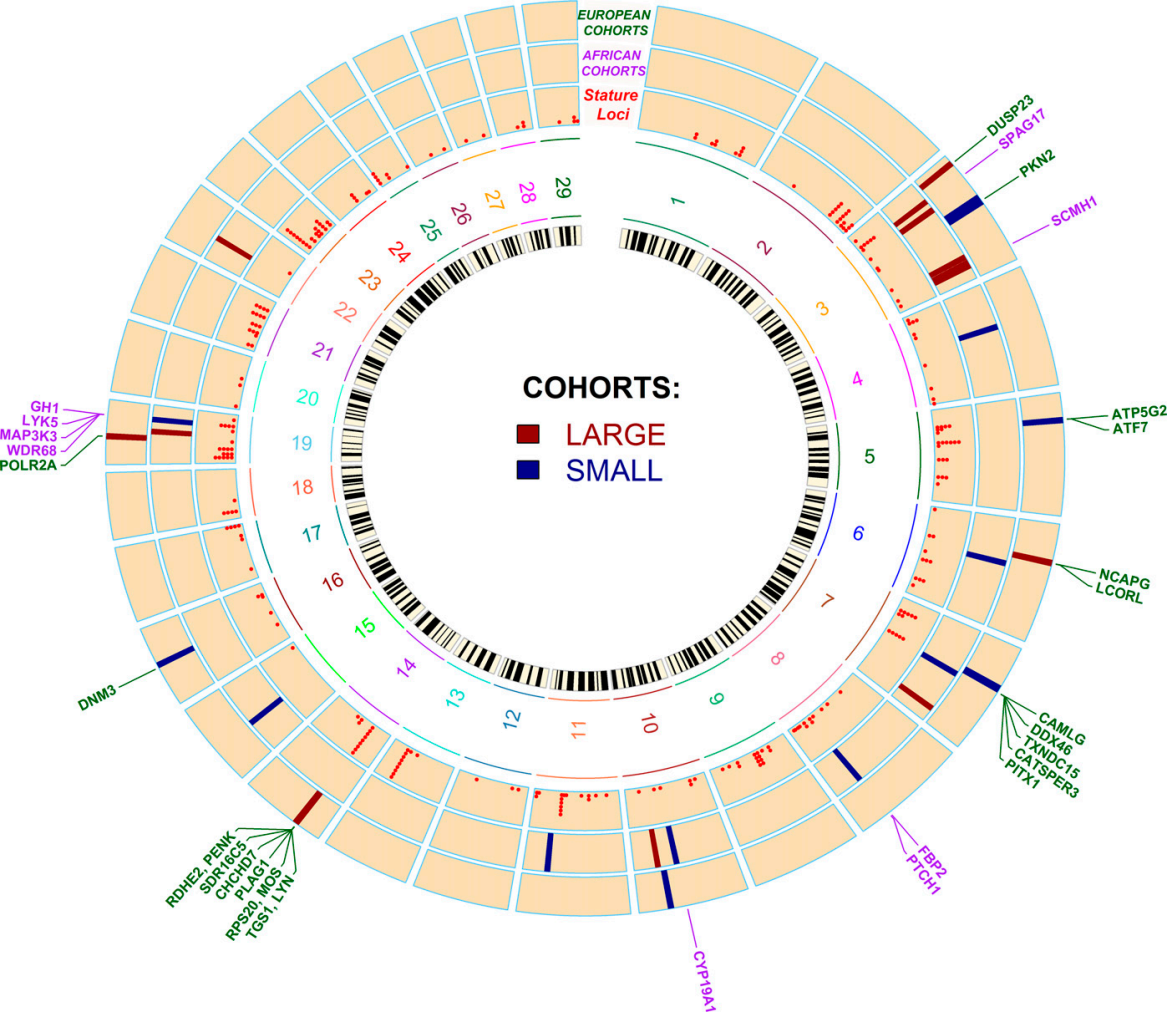
Circos image showing localization of genome-wide significant regions and candidate genes across all cohorts. The inner circle shows a bovine chromosome (29 autosomes) ideogram. The first outer circle, labeled as *Stature Loci*, shows the location of 134 loci listed in Table S4, and each red dot represents a stature-associated candidate gene (clustered red dots, within a locus, located on top of each other represent multiple neighboring candidate genes implicated in the same or different GWAS studies). Two circles, in the middle, labeled as *EUROPEAN COHORTS* and *AFRICAN COHORTS* show cohort-wise genomic regions identified by top 0.1% CSS (legends in the center for cohort-wise colored bars). The outer track shows genomic locations of 30 candidate genes within the 12 significant signatures of selection linked to bovine stature. Gene colors indicate whether they were identified in European (green) or African (purple) cattle types.

The Circos image (Figure 4) represents all 26 significant genomic regions, names of important candidate genes for bovine stature (Table 2), and the locations of all the orthologous genes from human GWAS (Table S4). Table 2 shows the list of those 12 genomic regions that harbor 30 candidate genes, which have previously been associated with stature in human GWAS and other species. The results from the major contrasting cohorts, especially large and small cohorts of European *Bos taurus*, supported the strategy of finding candidate gene regions by grouping phenotypically alike breeds. All the significant signatures of selection (four out of four) identified in the European large cohort contained known candidate genes in multiple species (Table 2) within region 1 (gene: *DUSP23*), region 6 (*NCAPG*, *LCORL*), region 10 (*TGS1*, *LYN*, *RPS20*, *MOS*, *PLAG1*, *CHCHD7*, *SDR16C5*, *RDHE2*, *PENK*), and region 11 (*POLR2A*). However, in cattle, only regions 6 and 10 have previously been investigated in relation to stature. In the European small cohort, three out of the four significant genomic regions, *i.e.*, region 3 (*PKN2*), region 5 (*ATP5G2*, *ATF7*), and region 7 (*CAMLG*, *DDX46*, *TXNDC15*, *CATSPER3*, *PITX1*) harbor candidate genes that were previously reported to be associated with human height.

**Table 2:** Significant selection regions that harbor candidate genes for stature identified by composite selection signals in all analyses of European and African cohorts

Region	BTA	Location (Mb)	Top 0.1 (1)% SNPs	Candidate Genes	Candidate Population	Source of Discovery (References)
1	3	9.35–10.06	1 (15)	*DUSP23*	European large	Human (Lanktree *et al.* 2011)
2	3	24.00–24.83	1 (9)	*SPAG17*	African large	Human (Weedon *et al.* 2008; Kim *et al.* 2010)
3	3	50.62–56.96	5 (47)	*PKN2*	European small	Human (Lanktree *et al.* 2011)
4	3	105.09–106.96	11 (30)	*SCMH1*	African large	Human (Weedon *et al.* 2008)
5	5	26.88–27.50	4 (8)	*ATP5G2*, *ATF7*	European small	Human (Okada *et al.* 2010)
6	6	38.29–39.75	10 (29)	*NCAPG*, *LCORL*	European large	Human, cattle, and horse (Gudbjartsson *et al.* 2008; Weedon *et al.* 2008; Soranzo *et al.* 2009; Lango Allen *et al.* 2010; Okada *et al.* 2010; Pryce *et al.* 2011)
7	7	43.42–46.75	20 (36)	*CAMLG*, *DDX46*, *TXNDC15*, *CATSPER3*, *PITX1**^a^*	European small	Human (Gudbjartsson *et al.* 2008)
8	8	83.05–83.89	3 (13)	*FBP2*, *PTCH1*	African small	Human and cattle (Weedon *et al.* 2008; Kim *et al.* 2010; Lanktree *et al.* 2011; Pryce *et al.* 2011)
9	10	59.38–59.97	1 (12)	*CYP19A1*	African small	Human (Okada *et al.* 2010; Lanktree *et al.* 2011)
10	14	24.79–28.25	21 (66)	*TGS1*, *LYN*, *RPS20*, *MOS*, *PLAG1*, *CHCHD7*, *SDR16C5*, *RDHE2*, *PENK*	European large	Human, cattle, and horse (Gudbjartsson *et al.* 2008; Soranzo *et al.* 2009; Okada *et al.* 2010; Lango Allen *et al.* 2010; Lettre *et al.* 2008; Pryce *et al.* 2011)
11	19	27.64–28.63	6 (16)	*POLR2A*	European large	Human (Lanktree *et al.* 2011)
12	19	48.01–49.01	1 (14)	*WDR68*, *MAP3K3*, *LYK5*, *GH1*	African small	Human (Gudbjartsson *et al.* 2008; Soranzo *et al.* 2009; Lanktree *et al.* 2011)

a Candidate gene locus is localized slightly over 1 Mb in the surrounding of a significant region.

In the African cohorts, several significant signatures of selection regions did not contain known stature-associated candidate genes. Consequently, the percentage of selection regions with known candidate genes was lower, *i.e.*, 29.4%, which is almost one-third of that of European cohorts (77.8%). Despite that, this percentage in the African cohorts was almost three-times more than that of the likelihood of occurrence by chance. Nevertheless, we identified five candidate gene regions (2, 4, 8, 9, and 12) in African cohorts that contain important genes for stature, as reported by multiple human GWAS (Table 2).

Finally, Figure S5 compares the distribution of CSS and its component tests (*F*_ST_, ΔDAF, and XP-EHH) at the 12 candidate regions harboring stature-associated genes. These comparisons show that the CSS scores are not being derived from one particular component selection test. The results also suggest that the three component tests provide complementary evidence to each other at the candidate regions. However, it is evident that many of these candidate regions would not have been detected with any of the component selection tests, particularly regions 1, 2, 5, 8, 9, and 12. These results provide overwhelming support for the CSS strategy, suggesting that it is a robust and efficient method for combining complementary evidence to detect the historical signatures of selection for complex traits, which otherwise cannot be detected by available tests of selection.

## Discussion

### CSS for complex traits

The CSS approach demonstrates that the test is generally applicable for the identification of trait-specific genomic regions for complex traits by comparing phenotypically contrasting populations. The CSS test combines multiple pieces of evidence from the rank distribution of different selection tests in a weighted index of signatures of selection. The power of the test and its constituents (*F*_ST_, ΔDAF, and XP-EHH) depends on the phenotypic and genetic divergence between the candidate and reference populations, as we demonstrated with simple binary monogenic (Randhawa *et al.* 2014) and complex polygenic traits (this study).

Use of multibreed cohorts has increased the discovery of trait-specific regions because of shared linkage disequilibrium between the causal mutations and neighboring SNPs (Kemper and Goddard 2012) arising from long-term historical selection. Contrasting patterns of genomic variation at the putative regions under selection across groups increases the likelihood of capturing the signatures of selection linked to stature. In our approach, signals of selection are amplified at candidate regions across breeds within groups, whereas background noise (false-positive signals) is reduced in the rest of the genome. Such noise is usually expected from the demographic history of breed formation and random genetic drift (Seichter *et al.* 2012). Overall, application of the CSS method combined with grouping breeds according to their wither height identified candidate regions in the bovine genome for this complex trait.

Detection of signatures of selection is valuable in the discovery of potential genomic regions of functional mutations affecting quantitative traits (Hayes *et al.* 2008). Recent investigations suggest that, in the absence of classic selective sweeps, signatures of selection for complex traits are less likely to be detected by using individual selection tests on single breed data (Kemper *et al.* 2014). Our approach combines multiple selection tests and grouping of phenotypically alike breeds, and has been found to be highly efficient at detecting signatures of selection and identifying candidate gene regions for complex traits. This approach makes use of existing resources under long-term historical selection and provides a relatively inexpensive entry for more detailed follow-up studies in the genetic architecture of complex traits.

### Detection of signatures of selection for bovine stature

In this study, we used bovine wither height (stature) measures from an online resource, namely the FAO database (DAD-IS 2014), recorded as averages for both males and females across multiple countries. Height and body size have generally been considered important traits in cattle during domestication. During early stages of domestication, selection favored animals with lower stature compared to wild ancestors (Karim *et al.* 2011). However, during the recent past, strong selection for increased stature has been applied in selected breeds of modern cattle (Ajmone-Marsan *et al.* 2010).

We found 26 bovine genomic regions in European and African *Bos taurus* carrying distinctive signatures of selection. Of these, 12 (46%) contained 30 stature-associated candidate genes derived from comparative mapping. We showed that when cohorts of randomly permuted animals were tested, a much lower proportion (8.3%) of the significant regions contained candidate genes, which is consistent with random expectations (∼7%). This suggests that our approach was powerful in capturing bovine genomic regions related to stature. Several of these should be considered novel because they were not previously reported in cattle.

It is noteworthy why different genomic regions are detected in the large and small cohorts within European and African cattle. The cohorts within each cattle type were compared against each other to compute the constituent (*F*_ST_, ΔDAF, and XP-EHH) selection tests of CSS. Two selection tests (ΔDAF and XP-EHH) provide directional signatures of selection. This implicates that different gene variants are regulating stature in large and small breeds of cattle (Visscher and Goddard 2011).

Several traits, such as birth weight, growth rate, adult weight, and stature, are found to be correlated with each other and have pleiotropic effects. Some of the genes in the validation regions (see below) have been previously found to be associated with multiple correlated traits. The FAO data on stature have been used to objectively classify cohorts of cattle, and the data were not adjusted for other traits given the nonavailability of FAO records for the correlated traits. Multibreed classification is expected to minimize the breed sampling effects; however, some of the candidate regions may not be directly reflecting selection of stature but could be confounded by some of the other correlated traits.

The significant CSS outside the candidate gene regions (Table S5 and Table S6) may indicate putative genes associated with height or targets of selection for traits other than stature (Utsunomiya *et al.* 2013; Druet *et al.* 2013; Ramey *et al.* 2013; Stella *et al.* 2010; Kemper *et al.* 2014; Perez *et al.* 2014; Porto-Neto *et al.* 2013; Rothammer *et al.* 2013; Gautier *et al.* 2009). Moreover, several additional significant CSS regions in African cattle have not been reported previously, given the limited reports on selective sweep analyses in the African breeds.

### Validation of known stature-associated genomic regions in cattle

This study validated two important candidate gene regions (regions 6 and 10) linked to stature in European breeds of cattle. Region 6 harboring *NCAPG* and *LCORL* genes located on BTA-6 has frequently shown strong signatures of selection in multiple breeds of cattle (Barendse *et al.* 2009; Druet *et al.* 2013; Flori *et al.* 2009; Gibbs *et al.* 2009; Kemper *et al.* 2014; Mancini *et al.* 2014; Pintus *et al.* 2013; Porto-Neto *et al.* 2013; Rothammer *et al.* 2013; Stella *et al.* 2010; Qanbari *et al.* 2014; Perez *et al.* 2014). Region 10 harboring *PLAG1* and *CHCHD7* genes on BTA-14 has been associated with height in humans (Gudbjartsson *et al.* 2008; Soranzo *et al.* 2009; Okada *et al.* 2010; Lettre *et al.* 2008; Lango Allen *et al.* 2010) and stature in cattle (Nishimura *et al.* 2012; Pryce *et al.* 2011; Karim *et al.* 2011; Fortes *et al.* 2013; Bolormaa *et al.* 2014). This locus was also identified as a candidate region based on selective sweeps in several cattle breeds that have been under strong selection for body size (Druet *et al.* 2013; Ramey *et al.* 2013; Kemper *et al.* 2014).

### Genomic regions associated with stature in European *Bos taurus*

Based on strong recent selection for stature in European *Bos taurus*, our comparison of the large cohort with the small cohort successfully localized height-related genes at all significant regions (regions 1, 6, 10, and 11). Regions 1 and 11 can be considered novel and may contain functional variants contributing to the genetic control of bovine stature, because they harbor *DUSP23* (BTA-3: 10.02-10.03 Mb) and *POLR2A* (BTA-19: 27.78-27.80) as candidate genes, respectively (Lanktree *et al.* 2011).

There can be some advantages of limiting body size in smaller breeds, for instance, in relation to ease of calving or resource-limited production systems. As noted above, early domestication favored selective breeding to reduce body size for ease of management (Karim *et al.* 2011; Ajmone-Marsan *et al.* 2010). With very few exceptions (*e.g.*, Dexter), recent history of European *Bos taurus* breeds do not document selective breeding for smaller size. Interestingly, 60% of all significant regions can be considered novel in the small cohort and harbor stature-associated genes, namely *PKN2* at region 3 (Lanktree *et al.* 2011), *ATP5G2* and *ATF7* at region 5 (Okada *et al.* 2010), and *CAMLG*, *DDX46*, *TXNDC15*, *CATSPER3*, and *PITX1* at region 7 (Gudbjartsson *et al.* 2008). The functional role of these candidate genes in relation to stature is not well-documented.

### Genomic regions associated with stature in African *Bos taurus*

Five candidate regions (2, 4, 8, 9, 12) in the African cohorts harbor nine candidate genes associated with human height identified by GWAS (Gudbjartsson *et al.* 2008; Soranzo *et al.* 2009; Okada *et al.* 2010; Lanktree *et al.* 2011; Kim *et al.* 2010; Weedon *et al.* 2008). Previously, *FBP2* and *PTCH1* genes underlying region 8 (BTA-8) have been associated with stature in European dairy and beef breeds, respectively (Weedon *et al.* 2008; Kim *et al.* 2010; Lanktree *et al.* 2011; Pryce *et al.* 2011). In region 12 (BTA-19), the growth hormone genes (*GH1*, *GH2*) are strong candidates for dairy production traits (Hayes *et al.* 2009; Rothammer *et al.* 2013; Mullen *et al.* 2010). Moreover, three genes (*WDR68*, *MAP3K3*, and *LYK5*) neighboring growth hormone genes have also been associated with human height in multiple GWAS reports (Gudbjartsson *et al.* 2008; Soranzo *et al.* 2009; Lanktree *et al.* 2011). The remaining three candidate regions, *i.e.*, regions 2, 4, and 9, harbor the stature-associated *SPAG17*, *SCMH1*, and *CYP19A1* genes, respectively (Weedon *et al.* 2008; Kim *et al.* 2010; Okada *et al.* 2010; Lanktree *et al.* 2011).

Overall, four of the five candidate regions identified in the African cohorts are novel for their association with bovine stature. This suggests that the strategy of combining CSS and multibreed panels showed utility in the African cohorts also, although the overall proportion of candidate gene regions detected was lower compared to European cohorts. High levels of *Bos indicus* admixture, smaller sample size, and ascertainment bias of the Illumina BovineSNP50 chip in the African *Bos taurus* breeds may have impacted the power to detect regions under selection (Randhawa *et al.* 2014; Decker *et al.* 2009; Gautier *et al.* 2010; Gautier and Naves 2011).

### Comparison of significant CSS regions between European and African *Bos taurus*

No candidate gene regions were found in common across the European and African cattle types. This may be due to the long-term differences in natural and production-oriented selective pressures in these diverse genetic archetypes (Edea *et al.* 2014; Gautier *et al.* 2009; Gautier and Naves 2011; Gibbs *et al.* 2009; Randhawa *et al.* 2014). Recent attempts to find the transferability of height-associated loci across human populations have shown comparable results (low consistency) for finding the same genetic variants when discoveries made in non-African ethnicities were compared in populations of African ancestry (Kang *et al.* 2010; Shriner *et al.* 2009). Our results indicate that there may be a persistent trend of dissimilarities for African genealogies across species, and some environmental factors would have shaped a comparatively different genetic architecture. We suggest that investigation of several other African breeds with sufficient sample sizes can further elaborate the nature of different candidate gene regions.

A region on BTA-13 harboring *UQCC* and *GDF5* genes, which we putatively linked to bovine stature (Randhawa *et al.* 2014), was also not detected in this study. The absence of BTA-13 genes (*UQCC* and *GDF5*) as the possible candidate for bovine stature in this study are due to the sensitivity of CSS (and its constituent tests of selection) to the sample composition because the cohort composition between the two papers is quite different regarding inclusion and exclusion of several breeds. Moreover, another gene, *ASIP* (related to coat color), was also located at the BTA-13 peak, and *ASIP* may also be another plausible candidate of the signatures of selection detected in the previous study.

In comparison to previous findings in cattle, we note that some of the known genes and regions related to bovine stature were not detected here, mainly because they were identified in *Bos indicus* and composite breeds (Pryce *et al.* 2011). Moreover, genetic architecture of stature is not considered fully analogous in cattle and human; our results suggest that many common genes might be participating in the physiological control of stature in the two species (Pryce *et al.* 2011).

Characterization of biological function of candidate genes could help our understanding of mechanisms underlying stature diversity. Future research should focus on regional genomic sequencing (involving multiple genes) rather than single gene-centric approaches. In addition, gene networks and functional pathway analyses on subsets of genes identified by genome-wide association or selection scans can help uncover the biological processes in complex traits.

## Conclusion

This study demonstrates an approach to detect signatures of selection for complex traits. We successfully used CSS in the analysis of the genetic architecture of a complex polygenic trait using multibreed panels. Our results demonstrated the localization of genomic regions harboring candidate genes of known effect related to body development, skeletal growth, and stature in cattle. We identified 26 regions with strong signatures of selection for stature, of which 12 regions contained known genes linked to height or stature and 9 could be considered novel in European and African *Bos taurus*. These results should be of interest for future investigations to characterize causal variants to explain their functional role in the diversity of bovine stature. Implementation of new tools, such as CSS, can resolve the selected genomic regions underlying the complex traits in livestock species. We suggest that the application of genome-wide selection scans and GWAS using larger resource populations with phenotypic information can further improve the fine-mapping of causal mutations controlling complex traits.
